# ‘Up with the LRRK’: a phosphorylated Rab10 assay for evaluation of LRRK2 activity and inhibitor engagement

**DOI:** 10.1042/BCJ20160671C

**Published:** 2016-09-12

**Authors:** Patrick A. Eyers

**Affiliations:** Department of Biochemistry, Institute of Integrative Biology, University of Liverpool, Liverpool L69 7ZB, U.K.

**Keywords:** inhibitor, knockout mice, LRRK2, Parkinson's disease, Phos-tag, Rab10

## Abstract

Protein kinases catalyse the addition of phosphate groups to Ser/Thr and Tyr residues in cognate substrates and are mutated or hyperactive in a variety of diseases, making them important targets for rationally designed drugs. A good example is the Parkinson's disease-associated kinase, leucine-rich repeat kinase 2 (LRRK2), which is mutated (and probably hyperactive) in a small, but significant, subset of patients. An exciting new approach for personalised therapy is the development of central nervous system (CNS)-active small-molecule kinase inhibitors, which could be employed to ‘normalise’ LRRK2 signalling in affected cell types. However, the development of such drugs requires validated assays for the analysis of target engagement and the assembly of a set of tools for interrogating LRRK2, and its substrates, both *in vitro* and *in vivo*. A new study published in the *Biochemical Journal* by Ito et al. establishes that a ‘Phos-tag’™-binding assay can be exploited to measure phosphorylation of a recently identified LRRK2 substrate (Ras-related protein in brain 10 (Rab10)), and to compare and contrast relative catalytic output from disease-associated LRRK2 mutants. Powerful *in vivo* chemical genetic approaches are also disclosed, in which the catalytic activity of LRRK2 is unequivocally linked to the extent of Rab10 phosphorylation and the effects of chemically distinct LRRK2 inhibitors are matched with on-target inhibition mechanisms mediated through LRRK2 and its substrate Rab10. These important findings should simplify the generic analysis of Rab10 phosphorylation in model biological systems and are likely to be applicable to other substrates of LRRK2 (or indeed other kinases) for which phospho-specific antibodies are either absent or unsatisfactory.

## Introduction

Protein phosphorylation is a reversible covalent modification that has the useful consequence of changing both the acidity (through introduction of negative charge) and the chemical nature of modified amino acid(s) [1]. These physiochemical changes can be exploited to measure the context, extent and lability of phosphorylation in vast number of ways, most classically through polyacrylamide gel-based quantification (typically western blotting and phospho-specific antibody procedures) or by using mass spectrometry (MS)-based approaches. However, although both MS and antibody strategies have become a standard methodology, phospho-specific antibodies are notoriously unreliable and are hard to quantify, while bottom-up MS often relies on robust detection of non-stoichiometric and multiple phosphorylated peptides in highly complicated mixtures. Both MS and antibody approaches also struggle to reveal complex phosphorylation ‘connectivity’ found in polypeptides [2], and it remains challenging to identify multiple phosphorylated residues that lie close together in primary sequence. To a first approximation, the extent of substrate phosphorylation reports the catalytic activity of protein kinase(s) [3]. Indeed, the development of kinase inhibitors as pharmaceutical agents relies on measuring ‘target engagement’ as part of the bench-to-bedside cycle, where quantification of the extent of substrate phosphorylation *in vitro* and *in vivo* in the presence of drug is of central importance [4]. However, discovering and validating physiological substrates of kinases remains highly relevant [5–7], in large part due to their emergence as critical biomarkers in disease biology.

## A generalised ‘Phos-tag’ solution for protein phosphorylation analysis?

**Figure 1.**
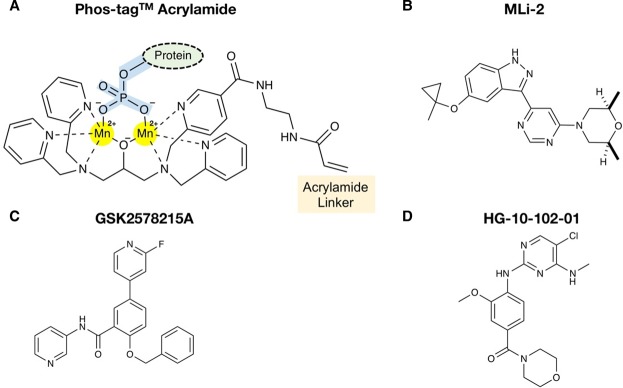
Chemical structures of Phos-tag cross-linking reagent and LRRK2 inhibitors. (**A**) General chemical structure of acrylamide Phos-tag reagent bound to a phosphate group that is itself covalently attached to a protein. The acrylamide becomes covalently cross-linked during gel polymerisation, and the divalent cation (typically Zn^2+^ or Mn^2+^) Phos-tag reagent is a high-affinity non-covalent affinity tag for phosphorylated proteins as they migrate through the gel. (**B**–**D**) Chemical structure of target-validated cell-permeable LRRK2 ‘probe compound’ inhibitors MLi-2 (**B**), GSK2578215A (**C**) and HG-10-102-01 (**D**).

‘A new study by Ito, Alessi and colleagues from GlaxoSmithKline (GSK) and The Michael J Fox Foundation for Parkinson’s Disease [8a] published in the Biochemical Journal reports an analysis of the Parkinsons' disease-associated kinase, leucine-rich repeat kinase 2 (LRRK2). Their work builds upon earlier seminal studies from Koike et al. [8] who developed a quantitative ‘Phosphate-binding tag’ (Phos-tag) approach to analyse peptide [9] and protein phosphorylation across a wide range of molecular masses [10,11]. Their solution was a modified SDS–PAGE procedure employing a stable Mn^2+^:phosphate:Phos-tag acrylamide complex (Figure 1A). This technology is also applicable to additional biological scenarios, where phosphate esters are relevant [12]. Notably, over the last decade, the Phos-tag approach has been exploited in over 500 published studies to ‘tag’ and then to evaluate phosphorylation-dependent changes in protein mobility using a simple modification of standard SDS–PAGE procedures. These protocols require the visualisation of retarded phosphorylated proteins, which is made possible by an alkoxide-bridged divalent metal ion (notably Mn^2+^) complex that is copolymerised in the gel prior to electrophoresis [10]. This approach builds upon earlier observations that changes in protein mobility imparted by phosphorylation (often, but not always, a reduction or ‘upward’ shift) can be detected after electrophoresis using a suitable method, typically dye binding or western blot with an antibody to the phosphorylated protein of interest. Indeed, subtle modifications of ‘standard’ SDS–PAGE procedures [13,14] were already known to exaggerate and permit the analysis of ‘abnormally’ migrating phosphoproteins in polyacrylamide gels [15–18].

## LRRK2 and a new link to vesicular trafficking

Continuing successes with kinase inhibitors in the oncology clinic have marked out protein kinases with atypical signalling profiles in other diseases, such as inflammation and neurological disorders, as potential targets for therapeutic intervention [19]. One such example is the LRRK2 protein kinase, a Ser/Thr kinase, which is activated by ‘hot spot’ autosomal-dominant R1441G or G2019S mutations found in ∼5% of Parkinson's disease sufferers [20]. Development of brain-penetrant modulators of hyperactive LRRK2 is hypothesised to be a useful way to normalise (decrease) LRRK2 signalling in dopaminergic neurons, and although several tool compounds have been developed, including the brain-penetrating compound HG-10-102-01 [21,22], LRRK2 inhibitors have yet to be clinically validated. Moreover, accurately measuring LRRK2 activity, substrate phosphorylation and compound target engagement in a rapid and efficient manner is an important challenge. This is on top of the knowledge that many, if not all, kinase inhibitors bind to more than one kinase. Therefore, ‘gold standard’ target validation requires the exploitation of multipronged approaches involving the analysis of chemically distinct compounds and deployment of drug-resistant kinase alleles [23–27]. Both these avenues are explored in the new *Biochemical Journal* study, which builds upon a recent ground-breaking phosphoproteomic study that uncovered Rab GTPase family members as new substrates (and potential biomarkers) for LRRK2 [18]. This work opens up the study of new signalling mechanisms connecting phosphorylation with vesicular cell trafficking in both normal and disease states. Interestingly, distinct Rab family members have also been recently shown to be phosphorylated by the mitochondrial membrane protein kinase termed PTEN-induced putative kinase 1 (PINK1), which is itself also implicated in Parkinson's disease [28]. Getting to grips with mechanistic aspects of Rab10 regulation by LRRK2 therefore remains an important new challenge.

## New approaches to analyse LRRK2 and its novel substrate Rab10

In their recent study [8a], Ito et al. demonstrate unequivocally that in cells LRRK2 phosphorylates Rab10 at a Thr residue (Thr73 in vertebrates) that is widely conserved in the effector binding ‘switch-II’ motif of Rab family members. The LRRK2 substrate specificity was initially defined several years ago [20], establishing it as a context-specific Thr-favouring kinase, whose substrates include the cytoskeletal protein moesin [5]. Hot on the heels of these discoveries [6,29], the Dundee group have now developed and validated a Phos-tag gel-based assay for LRRK2-mediated phosphorylation in multiple cell-based systems [8a]. Indeed, by combining Phos-tag acrylamide Mn^2+^-based reagents (synthesised in-house) with commercial Rab10 monoclonal antibodies [30], the LRRK2 reagent ‘toolbox’ now appears to have reached a critical mass that will support LRRK2 analysis in a variety of biological systems and organisms. In their new paper, Ito et al. also confirmed the LRRK2-catalysed appearance of a higher, faster migrating phosphorylated form of Rab10 (the ‘up’ of the title of this commentary) containing Rab10 phosphorylated on Thr73 in Phos-tag gels.

Further work exploiting the Phos-tag system demonstrated that HA-tagged Rab10 phosphorylated by overexpressed R1441G LRRK2 also led to significant retardation of Rab10 in cells. These data were backed up *in vitro* using a pT73 Rab10 antibody, which also picked up the slower migrating Rab10 protein [8a]. Consistently, pre-incubation with the potent LRRK2 inhibitor cis-2,6-dimethyl-4-(6-(5-(1-methylcyclopropoxy)-1H-indazol-3-yl)pyrimidin-4-yl)morpholine (MLi-2) [21] blocked appearance of the retarded Rab10. Even more convincingly, expression of a T73A Rab10 mutant prevented the appearance of the slower migrating Rab10, suggesting that phosphorylation at this site is recognised by the Phos-tag cross-linking reagent. A key strength of the new study concerns the finding that the Phos-tag gel system can be employed to validate Rab10 as both a bona fide endogenous substrate of LRRK2, and for measuring relative LRRK2 activity and activation kinetics. Indeed, the deletion of LRRK2 from mouse embryonic fibroblasts (MEFs), expression of a ‘dominant-negative’ kinase dead LRRK2 allele designed to prevent ATP:Mg binding (D2017A), or prior cellular incubation with three chemically diverse LRRK2 inhibitors (Figure 1B–D), all blocked the appearance of the retarded Rab10 [8a].

## ‘Drug resistance’ approaches to analyse LRRK2 inhibitors

An impressive validation of the Rab10 Phos-tag technique also comes from the finding that expression of a ‘drug-resistant’ (A2016T) allele of LRRK2 decreased the ability of the LRRK2 inhibitor MLi-2 to block the appearance of the slower migrating Rab10 phosphorylated protein. Indeed, the IC_50_ value shift for MLi-2 of some two orders of magnitude between wild-type LRRK2 and A2016T LRRK2-expressing MEFs represents unequivocal evidence that LRRK2 and Rab10 are a physiological kinase substrate pair in this system [8a]. Further *in vivo* evidence also comes from a mouse lung analysis, where a D2017A knockin blocked Rab10 phosphorylation, and an A2016T knockin [6] uncoupled the effects of MLi-2 *in vivo*. Such approaches are very powerful for evaluating the effects of this and other target-validated LRRK2 drug-like compounds, in pathological models of LRRK2 signalling.

An important new analytical use for Phos-tag gels pertaining to Rab10 phosphorylation was also described in the new study. Initially, the authors provided evidence that MEF cells derived from knockin mouse models expressing Parkinson's-associated R1441G or G2019S LRRK2 mutants exhibit increased levels of retarded (phosphorylated) Rab10 compared with control mice when analysed using Phos-tag gels with appropriate small-molecule controls. Complimentary phospho-specific antibodies that recognise pS935 LRRK2 did not mirror this trend, probably because this phosphorylation site is under the complex control of other kinase(s); in the case of the R1441G LRRK2 mutant, this site did not show any change in phosphorylation [8a]. It is tempting to speculate that the Phos-tag approach is superior to current phospho-specific antibody-mediated analysis using LRRK2, especially in the context of pathological LRRK2 mutations. This is strongly supported by an important new kinetic analysis of the dephosphorylation of Rab10 as measured by Phos-tag SDS–PAGE. Here, the very rapid dephosphorylation of Rab10 evident from Phos-tag analysis in response to a panel of LRRK2 inhibitors was not mirrored by analysis using a pS935 LRRK2 antibody. Indeed, the kinetics of LRRK2 dephosphorylation were markedly slower using this assay (up to 40 fold more so than Rab10). These data, which were itself internally validated with a ‘drug-resistant’ G2019S LRRK2 mutant and the compound GSK2578215A [31], might be useful for evaluating the appropriate ‘window’ of target engagement for other LRRK2 inhibitors *in vivo* and might have a profound effect on the choice of selection of Phos-tag Rab10 assays over LRRK2 phospho-specific antibodies, especially under conditions where Rab10 phospho-specific antibodies (such as Rab10 pT73) are ineffective or limiting.

## Conclusion

Cell signalling researchers are working in a golden age of chemical biology. Guided by probe and clinical compound candidates emerging from enlightened pharmaceutical companies and supported by commercial enterprises lying at a new interface between industrial and academic research, the traditional lack of access to small molecules is no longer a major impediment to research progress. Arguably, scientists are now challenged with new sets of issues, led by the twin problems of compound target validation (does a compound actually engage the expected target in the system of interest?) and target specificity (can a compound be employed to rule-in/rule-out a protein in a specific event?). Although phosphorylation site mapping has been revolutionised by proteomics and mass spectrometry workflows, most day-to-day experiments still rely on workhorse approaches for evaluating the activity of protein kinases, usually through indirect analysis of their substrates. Of course, protein phosphorylation can still be established and quantified using classical biochemistry approaches (e.g. cellular ^32^P labelling and Edman degradation) and ^32^P-based kinase assays [32] are still employed relatively widely. However, the vast majority of researchers regularly turn to antibody-based read-outs for evaluating intracellular substrate phosphorylation, taking advantage of the ‘unique’ consensus of a phosphorylated epitope to generate (and purify) phospho-specific antibodies. However, since a million or so phospho-specific antibodies might be needed to evaluate all individual phosphosites in cells [33], generic Phos-tag approaches provide a distinct quantitative option for phosphoprotein analysis.

The study by Ito et al. [8a] also represents a chemical biology *tour-de-force*; since it elegantly exploits drug-resistant LRRK2 alleles in a cellular context to validate that cellular effects of such compounds are derived from a direct interaction with LRRK2, and not an unintended kinase, or kinases [34]. A central strength of the Phos-tag-bound Rab10 assay is that phosphorylated amino acids in Rab10 do not need to be mapped (a weakness of phospho-specific antibodies, which require this information, *a priori*). Instead, electrophoretic retardation simply reflects phosphorylated Rab10 that is readily accessible to the Phos-tag affinity reagent. This technique might therefore also be useful in other scenarios where analysis of kinase and substrate pairings need to be either quantified, or is not well-served by phospho-specific antibodies. Given the findings of Ito et al. examining LRRK2 and Rab10, it will be very interesting to conduct a side-by-side comparison of Phos-tag derived and phospho-specific antibody data for a large panel of kinases and their substrates. This significant undertaking might even change the way we analyse or report the effects of disease-associated mutations on their substrates in the future, as could be the case for LRRK2 in both Parkinsonian models and stratified patients.

## Abbreviations

CNS, central nervous system; LRRK2, leucine-rich srepeat kinase 2; MEFs, mouse embryonic fibroblasts; MLi-2,cis-2,6-dimethyl-4-(6-(5-(1-methylcyclopropoxy)-1H-indazol-3-yl)pyrimidin-4-yl)morpholine; MS, mass spectrometry; Phos-tag, Phosphate-binding tag; PINK1, PTEN-induced putative kinase 1; Rab10, Ras-related protein in brain 10.

## Competing Interests

The Author declares that there are no competing interests associated with this manuscript.
